# Controlling for Artifacts in Widefield Optical Coherence Tomography Angiography Measurements of Non-Perfusion Area

**DOI:** 10.1038/s41598-019-43958-1

**Published:** 2019-06-24

**Authors:** Lucas R. De Pretto, Eric M. Moult, A. Yasin Alibhai, Oscar M. Carrasco-Zevallos, Siyu Chen, ByungKun Lee, Andre J. Witkin, Caroline R. Baumal, Elias Reichel, Anderson Zanardi de Freitas, Jay S. Duker, Nadia K. Waheed, James G. Fujimoto

**Affiliations:** 1 Department of Electrical Engineering and Computer Science, Research Laboratory of Electronics, Massachusetts Institute of Technology, Cambridge, Massachusetts USA; 2 Nuclear and Energy Research Institute IPEN-CNEN/SP, São Paulo, Brazil; 3 New England Eye Center, Tufts Medical Center, Boston, Massachusetts USA

**Keywords:** Imaging and sensing, Retinal diseases

## Abstract

The recent clinical adoption of optical coherence tomography (OCT) angiography (OCTA) has enabled non-invasive, volumetric visualization of ocular vasculature at micron-scale resolutions. Initially limited to 3 mm × 3 mm and 6 mm × 6 mm fields-of-view (FOV), commercial OCTA systems now offer 12 mm × 12 mm, or larger, imaging fields. While larger FOVs promise a more complete visualization of retinal disease, they also introduce new challenges to the accurate and reliable interpretation of OCTA data. In particular, because of vignetting, wide-field imaging increases occurrence of low-OCT-signal artifacts, which leads to thresholding and/or segmentation artifacts, complicating OCTA analysis. This study presents theoretical and case-based descriptions of the causes and effects of low-OCT-signal artifacts. Through these descriptions, we demonstrate that OCTA data interpretation can be ambiguous if performed without consulting corresponding OCT data. Furthermore, using wide-field non-perfusion analysis in diabetic retinopathy as a model widefield OCTA usage-case, we show how qualitative and quantitative analysis can be confounded by low-OCT-signal artifacts. Based on these results, we suggest methods and best-practices for preventing and managing low-OCT-signal artifacts, thereby reducing errors in OCTA quantitative analysis of non-perfusion and improving reproducibility. These methods promise to be especially important for longitudinal studies detecting progression and response to therapy.

## Introduction

Over the past five years, optical coherence tomography angiography (OCTA)^1,2^, a functional extension of optical coherence tomography (OCT), has emerged as a promising modality for visualizing and measuring ocular vasculature. Compared to dye-based angiography, such as fluorescein angiography (FA) or indocyanine green angiography (ICGA), OCTA has several advantages: because OCTA is derived from OCT, intrinsically co-registered structural data are available for concurrent viewing; it is rapid and non-invasive, enabling imaging at each patient visit and in situations in which FA/ICGA may not be indicated; dye-free imaging enables clear visualization of the retinal and choriocapillaris vasculatures without obscuring fluorescence from dye leakage while also eliminating risk of allergic reactions; and depth resolution allows separate visualization of vascular plexuses and localization of vasculature to anatomical layers. Nevertheless, OCTA also has important limitations: it does not directly visualize leakage; it involves a complex set of image processing steps, leaving it vulnerable to artifacts; it requires acquisition of repeated B-scans to detect motion contrast from flowing blood, necessitating high imaging speeds and limiting fields-of-view; and is currently higher cost than OCT.

At the time of this writing, the large majority of studies involving commercial OCTA use 3 mm × 3 mm or 6 mm × 6 mm fields-of-view, much smaller than the fields-of-view afforded by standard FA/ICGA imaging. However, recent developments in commercial OCTA permit larger, 12 mm × 12 mm, fields-of-view, facilitating OCTA assessment of vasculature beyond the macula. This capability is promising for a number of applications, including assessment of non-perfusion area in diabetic retinopathy, where FA/ICGA studies have reported that peripheral non-perfusion may occur prior to macular non-perfusion^3–5^. Indeed, OCTA studies with commercial systems^6,7^ and modified commercial systems^8–11^ reported large non-perfused regions in the periphery of eyes of diabetic patients. However, achieving reliable widefield OCTA imaging is more challenging—both technically and procedurally—than imaging of smaller fields-of-view, and such wide field images can give rise to artifacts that make images more difficult to interpret. Moreover, artifacts make quantitative measurements less reliable, limiting the clinical utility of widefield OCTA metrics, such as non-perfusion area, and making the technology less efficacious for disease detection, monitoring of disease progression, and evaluation of therapy response. Minimizing the effects of artifacts, and thereby improving measurement repeatability, is especially important for longitudinal studies, where artifact-induced variations can obscure the statistical significance of findings, necessitating larger patient enrollments and/or follow-up periods.

Although several artifacts complicate widefield OCTA imaging, low-OCT-signal artifacts become increasingly severe with increasing fields-of-view. With this in mind, the aims of this study are fourfold: first, to describe the *causes* of low OCT signal in widefield imaging; second, to investigate the *effects* of low OCT signal on OCTA images; third, to develop *solutions* for managing artifacts in widefield OCTA; and fourth, to describe best-practices for *prevention* of artifacts in widefield OCTA. These four aims are discussed in *Parts I-IV* of this study, respectively.

## Methods

### Patient selection

The study was approved by the Institutional Review Boards at Tufts Medical Center and the Massachusetts Institute of Technology. This research adhered to the tenets of the Declaration of Helsinki and complied with the Health Insurance Portability and Accountability Act of 1996. Written informed consent was obtained prior to imaging. This study includes retrospectively selected data from diabetic patients who were imaged at the retina clinic at the New England Eye Center, Boston, between January 2017 and July 2017. Each subject received a dilated fundus examination, color fundus photography, structural OCT imaging and, if clinically indicated, FA. Using the Early Treatment of Diabetic Retinopathy Study (ETDRS) standard grading protocol, diabetic retinopathy (DR) was classified as: no DR, mild non-proliferative DR (NPDR), moderate NPDR, severe NPDR, or proliferative DR (PDR).

### OCTA imaging

All subjects underwent OCTA imaging with a 100 kHz swept source OCTA (SS-OCTA) instrument (PLEX Elite 9000, Carl Zeiss Meditec Inc., Dublin, CA) with a ~1050 nm central wavelength, a ~6.3 µm axial resolution, and a ~20 µm lateral resolution. Volumes covering a 12 mm × 12 mm field-of-view, and centered at the fovea, were acquired. Each 12 mm × 12 mm volume consisted of 500 A-scans per B-scan (24 µm spacing between adjacent A-scans) and 500 B-scan locations per volume (24 µm spacing between adjacent B-scans); two repeated B-scans were acquired at each B-scan location for OCTA processing. FastTrac motion correction (Carl Zeiss Meditec, Inc., Dublin, CA), which detects and tracks eye motion via line scan ophthalmoscope (LSO) fundus imaging, was used during all acquisitions. *En face* OCTA images of the full retinal vasculature were formed using the PLEX Elite 9000’s default automatic segmentation algorithm. All OCTA imaging was performed by trained ophthalmic photographers who, if required, repeated imaging several times to ensure acquisition of images with good OCT signal level and minimal motion artifacts. This practice is consistent with the standard imaging protocol at the New England Eye Center.

#### Part I: causes of low OCT signal in widefield OCT

Because OCTA data are derived from their underlying OCT data, an understanding of the causes of low OCT signal in widefield *OCT* is required to understand low-OCT-signal artifacts in widefield *OCTA*. For this, it is helpful to recall some pertinent aspects of OCT imaging:

1. OCT imaging employs beam scanning, wherein A-scans are sequentially acquired as the OCT beam is scanned over the field-of-view. Beam scanning differs from full-field imaging, such as fundus camera imaging, where the entire field-of-view is acquired simultaneously, without scanning.
2. OCT instruments scan the OCT beam using a pair of perpendicular scanning mirrors arranged such that rotation of one mirror scans the beam along the horizontal axis, and rotation of the other mirror scans the beam along the vertical axis.
3. With proper system alignment, the pivot positions of the scanning mirrors (i.e., the positions about which the mirrors rotate) are optically relayed to the patient’s pupil plane (i.e., the plane containing the pupil) so that mirror rotation changes the angle between the OCT beam and pupil plane, but does not change the transverse position of the OCT beam within the pupil. This requires the instrument to be aligned at a specific working distance from the eye (see Fig. 1); incorrect alignment causes the OCT beam to change transverse position within the pupil as the scanning mirrors are rotated which increases the likelihood of vignetting.

**Figure 1:**
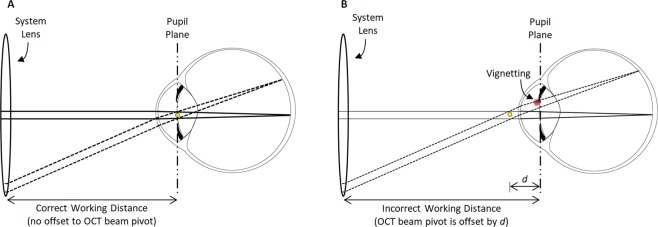
Illustration of how widefield OCT(A) increases susceptibility to axial alignment error and thereby exacerbates vignetting. (**A**) With the correct working distance, the OCT beam pivot (yellow dot) is coincident with the pupil plane. (**B**) With an incorrect working distance, the OCT beam pivot is offset (here by a distance d) from the pupil plane. When the OCT beam is parallel (solid rays) to the optic axis of the eye, there is no vignetting in either situation. Moreover, with the correct working distance, there is no vignetting even when OCT beam scanned to a different position on the retina (dashed rays); this is because although the scanning changes the beam angle, it does not translate the beam at the pupil plane. However, with an incorrect working distance, when the OCT beam is scanned, the beam changes both its angle and transverse position at the pupil plane, the latter of which results in vignetting (red area). Since the amount of translation increases with increasing scan angles, larger OCTA fields-of-view increase the likelihood of vignetting, and make correct instrument alignment increasingly important. Methods for correct instrument alignment are discussed in Part IV.
4. The OCT beam is focused by a combination of the eye and system optics. As with any imaging system, there is a limited axial (depth) range over which the beam remains focused. In OCT, the confocal parameter, a standard measure of depth of field, is ~0.5 mm.
5. Light from the incident OCT beam is backscattered by retinal tissues, collected by the system, and then processed to generate the OCT image. Importantly, OCT uses “confocal” collection, which means that only backscattered light that comes from within the focal depth of field and retraces its incident path is collected. In OCT, confocal collection is achieved by emitting and collecting light with an optical fiber, which acts like a pinhole.
6. Fourier domain OCT (FD-OCT) systems use either spectral domain OCT (SD-OCT) or swept source OCT (SS-OCT) detection. SD-OCT uses spectrometers to detect the interference spectrum of the backscattered light from different axial depths; while SS-OCT uses swept light sources without requiring a spectrometer. Both these technologies impose limits on the axial imaging range: in SD-OCT the range is limited by the spectrometer resolution, and in SS-OCT the range is limited by the instantaneous line-width of the swept light source.

These basic features of OCT imaging—beam scanning, eye and system optics, limited focal range, confocal collection, and interferometric detection—provide a framework to understand the causes of low OCT signal in widefield imaging. Table 1, adopted from a detailed discussion by Kolb *et al*.^12^, summarizes common causes of low OCT signal, and discusses why some causes are exacerbated by widefield imaging.

**Table 1:** Common causes of low OCT signal, adopted and expanded from Kolb *et al*.^12^.

Cause of Low OCT Signal	Description	Increased Prevalence/Severity with Increased Field Size?
*Vignetting*	Vignetting occurs when the OCT beam clips the iris during scanning, reducing the light that is incident on the retina. Vignetting is further explored in Fig. 1.	*Yes*. As illustrated in Fig. 1, widefield scanning is more sensitive to alignment of the instrument’s working distance. Since standard OCT has two beam pivots, widefield scanning is also more sensitive to transverse alignment.
*Ocular aberrations*	Backscattered OCT light is collected by an optical fiber; ocular aberrations cause distortions that reduce the collected light.	*Yes*. Ocular aberrations are more pronounced when the OCT beam is at a higher angle of incidence^26^.
*System aberrations*	Aberrations occurring in the system optics have a similar effect as ocular aberrations, decreasing collection of backscattered light.	*Yes*. Optical aberrations in the instrument are typically more pronounced at larger scan angles. Moreover, existing OCT instruments may not use optical designs optimized for widefield imaging.
*Angle-dependent backscattering*	OCT detects light that is backscattered by ocular structures; however, the amount of backscattering depends on the angle of incidence.	*Yes*. Wide fields-of-view include regions of increased retinal curvature, which have a high angle relative to the OCT beam, and backscatter less.
*Retina moving out of focus*	Similar to aberrations, if the retina is not within the focal range, the collection of backscattered light is decreased.	*Yes*. Due to retinal curvature, in wider fields-of-view the distance of the retina changes appreciably, which can cause some regions to be out of focus.
*Signal roll-off*	SD-OCT has sensitivity roll-off^27^, causing structures farther from the system’s zero-delay to have a reduced OCT signal. SS-OCT has less sensitivity roll-off than SD-OCT.	*Yes*. As above, the larger range of axial distances increases the prevalence and severity of signal roll-off artifacts.
*Cataracts*, *vitreous opacities*, *intra/sub-retinal fluid*, *& hemorrhaging*	Any structures that scatter or absorb the incident and/or backscattered OCT beam reduce the detected OCT signal.	*No*. To the best of our knowledge, these factors do not change with wider fields-of-view, unless widefield scanning causes the OCT beam to intercept vitreous opacities.
*Vascular shadowing*	As above, light scattering and absorption from blood vessels reduces the light reaching underlying tissues.	*No*. To the best of our knowledge, vascular shadowing is not substantially increased with wider fields-of-view.
*Retinal pigment epithelium*	Because the retinal pigment epithelium (RPE) is highly absorbing and scattering, the OCT signal reaching underlying structures is reduced. This is particularly a concern in 840 nm wavelength imaging (e.g., commercially available SD-OCT systems)^28,29^	*No*. To the best of our knowledge, RPE-associated absorption and scattering effects are not substantially increased with wider fields-of-view.

Of the causes in Table 1, for moderate fields-of-view, such as the 12 mm × 12 mm fields of this study, vignetting typically predominates, and therefore merits further discussion. Vignetting occurs when the incident OCT beam is partially, or fully, blocked by the iris. While this blockage can be caused by a large incident beam and/or a small pupil, with standard beam sizes and a dilated pupil, vignetting is primarily caused by misalignment between the instrument and the eye. Misalignments can be transverse or axial: transverse misalignments cause a transverse offset of the OCT beam from the pupil center, and axial misalignments cause an axial offset between the OCT beam pivot and the pupil plane. Axial offsets, which are common in OCT because of incorrect working distance and/or patient movement, generate increasingly severe vignetting with increasingly wide scan angles (Fig. 1). Thus, widefield OCT(A) is more sensitive to axial alignment than smaller fields of view.

#### Part II: Effects of low OCT signals on widefield OCTA

Having established that widefield imaging is more likely to produce regions of low OCT signal, we turn our attention to understanding how low OCT signals produce OCTA artifacts. Throughout, we frame our discussion in terms of OCTA measurement of non-perfusion area.

### How low OCT signals influence OCTA processing and measurement

**Figure 2:**
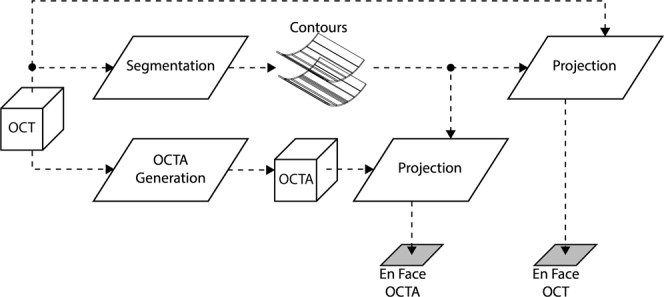
Schematic of processing steps used to generate *en face* OCT(A) images. The OCT volume is segmented, typically automatically, to form a set of contours. Usually the OCT signal, and not the OCTA signal, is used for segmentation (as shown here). The OCT and OCTA volumes are then projected (using averaging or histogram methods) between the segmented contours. This transforms volumetric (3-D) OCT(A) data into *en face* (2-D) OCT(A) data. Because the *en face* OCTA depends on the OCTA generation and segmentation steps, which in turn depend on the underlying OCT data, artifacts in the OCT data ultimately cause artifacts in the *en face* OCT(A) data.

Low OCT signals affect OCT data as well as all measurements that are derived from OCT data (Fig. 2). In particular, OCTA is computed from OCT data by detecting fluctuations between repeated B-scans caused by flowing blood cells. Since valid OCTA data cannot be obtained from regions with low or noisy OCT signals, these regions are removed by a process known as OCTA “thresholding”^13^. If a normalized OCTA algorithm is used, then a thresholding step is explicitly applied, which masks regions of low OCT signal. If, however, different OCTA algorithms which are not normalized are used, then regions of low OCT signal inherently generate regions of low OCTA signal; this, taken together with the image display, generates an implicit thresholding effect. Thus, regions with low OCT signal cause artifactual regions of low OCTA signal, irrespective of the presence/absence of blood flow.

Additionally, because most retinal vasculature lies in the plane of the retina, OCTA data are typically viewed as *en face* projections. To form an *en face* projection the OCT volume must be segmented to determine contours between which the OCTA volume is projected. However, regions of low OCT signals have a reduced contrast between different retinal layers, which often causes segmentations errors. Thus, low OCT signals can also affect *en face* OCTA images through segmentation errors. Summarizing, low OCT signals can produce two types of artifacts in *en fac*e OCTA images:

1. ***Thresholding Artifacts from Low OCT Signal:*** artifacts due to OCTA thresholding. In this type of artifact, low OCT signals directly result in invalid OCTA data.
2. ***Segmentation Artifacts from Low OCT Signal:*** artifacts due to segmentation errors. In this type of artifact, low OCT signals cause segmentation errors, which results in an inaccurate *en face* OCTA image.

### How low-OCT-signal artifacts affect OCTA interpretation

When an OCTA image is clinically interpreted, it is implicitly assumed that high OCTA signals (brighter pixels) imply blood flow, and that low signals (darker pixels) imply low/no blood flow. Without artifacts, the presence or absence of blood flow is the only determinant of the OCTA signal and our implicit assumptions hold. However, in this description we are neglecting the role of the interscan time, which determines the range of detectable blood flow speeds^14^. For the purposes of this study we assume the blood flow is sufficiently fast—and that the interscan time is sufficiently long—so as to be within the detectable OCTA range. This assumption holds for most commercial OCTA instruments which use long interscan times.

**Figure 3:**
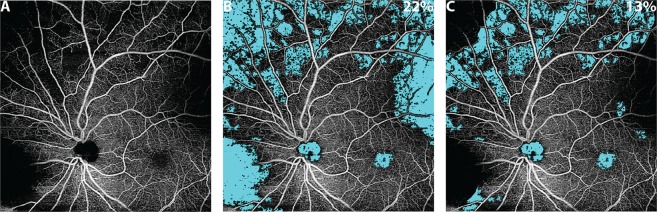
Effect of artifacts on the percentage non-perfusion area (PNPA) metric of a 12 mm × 12 mm OCTA image of the retinal vasculature of an eye with mild NPDR from a 68 year-old. (**A**) *En face* OCTA image showing many regions of low OCTA signal; from this *en face* OCTA image alone, it is unclear if all regions of low OCTA signal correspond to regions of actual low/no blood flow, or whether some are false positives caused by low-OCT-signal artifacts. (**B**) The uncorrected identification of non-perfusion areas (teal). (**C**) The corrected identification of non-perfusion areas, where regions of low-OCT-signal artifacts have been manually excluded (see Fig. 4). The PNPAs, given in the top right corner of panels (B,C), change from 22% (uncorrected) to 13% (corrected).

**Figure 4:**
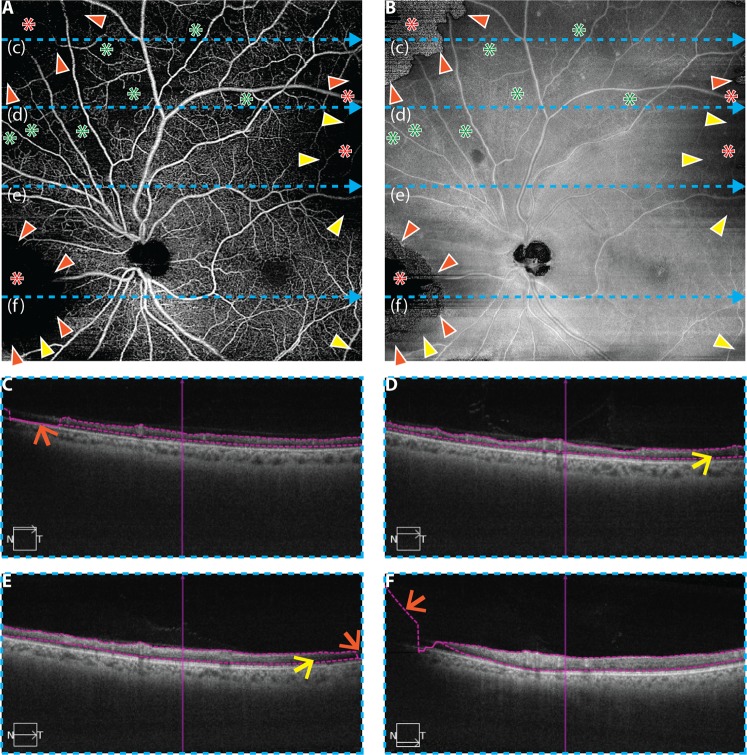
Illustration of cross-sectional, *en face*, and orthoplane approaches for artifact detection in an eye from a 68 year-old with mild NPDR; same eye as in Fig. 3. (**A**) 12 mm × 12 mm *en face* OCTA of retinal vasculature. (**B**) Corresponding *en face* OCT. (**C**–**F**) OCT B-scans extracted from the dashed blue lines labelled (c–f), respectively. In panels (**A**,**B**), the orange arrowheads point to the boundaries of segmentation artifacts (i.e., transitions from valid to invalid segmentation); note the abrupt change in the *en face* OCT at these locations (panel B). Yellow arrowheads point to the boundaries of thresholding artifacts; note the characteristic low signal in the *en face* OCT (panel B); red asterisks indicate regions of low OCTA signal caused by low-OCT-signal artifacts (either thresholding or segmentation artifacts), and green asterisks correspond to regions of low OCTA signal that correspond to true regions of low/no blood flow (i.e., areas of non-perfusion). In panels (C–F), orange arrows point to segmentation errors, and yellow arrows point to regions that generate thresholding artifacts as a result of low OCT signal. In the cross-sectional approach for artifact detection, only panels C–F are used; in the *en face* approach, only panels A and B are used; and, in the orthoplane approach, suspect regions are flagged using panels A and B, and cross-sectional OCT B-scans are taken through these locations (panels C–F).

Thresholding and segmentation artifacts affect the *en face* OCTA image such that it is not necessarily true that brighter OCTA pixels imply blood flow, nor that darker OCTA pixels imply low/no blood flow. Thus, careful attention is required to detect possible artifacts and exclude them from quantitative analysis. To appreciate that this is more than just a theoretical conundrum, consider the *en face* OCTA image of Fig. 3A. Do all the regions of low OCTA signal correspond to regions of low/no blood flow? The answer—revealed in Fig. 3C, and detailed in Fig. 4—is *no*.

### How low-OCT-signal artifacts affect quantitative OCTA metrics

Recognizing that low-OCT-signal artifacts complicate the interpretation of OCTA images, we now address how these artifacts affect quantitative metrics. To illustrate this concept, consider the percentage non-perfusion area (PNPA), a representative metric for assessing capillary loss:

1 $$Uncorrected\,PNPA=\frac{\text{Area of Low OCTA Signal}}{\text{Field} \mbox{-} \text{of} \mbox{-} \text{View Area}}\,\times \,\text{100\%}$$

Next, consider the “corrected” PNPA:

2 $$Corrected\,PNPA={\textstyle \tfrac{\text{Area of Low OCTA Signal}-\text{Area of Artifactual Low OCTA Signal}}{\text{Field} \mbox{-} \text{of} \mbox{-} \text{View Area}-\text{Artifact Area}}\times \text{100\%}}$$

Unlike the uncorrected PNPA, the corrected PNPA excludes regions affected by measurement artifacts. By comparing these two metrics, one can gain insight about how artifacts affect quantitative OCTA measurements. As an example, consider the uncorrected and corrected PNPAs of the widefield OCTA of Fig. 3A. In this paper, the uncorrected PNPA was measured using a custom algorithm that automatically identifies areas of low OCTA by a “flooding” segmentation of the spatial variance-transformed OCTA image; the reader is referred to Alibhai *et al*. for further details^15^. The reader should note that there are several other, previously published, algorithms that have been used to detect NPAs^16–20^. To reduce the influence of OCTA noise, the algorithm only considers non-perfusion areas larger than an empirically determined threshold of ~0.15 mm^2^ (250 contiguous pixels). The rationale for this threshold is the limited A-Scan sampling density of current widefield OCTA images, which causes smaller intercapillary areas to be poorly resolved and prone to erroneous segmentations. Specifically, 0.15 mm² is an estimate of the area of the smallest circular NPA that can be reliably detected^15^. Importantly, the algorithm implicitly assumes that an area of low OCTA signal corresponds to an area of non-perfusion (i.e., it does not incorporate the possibility of low-OCT-signal artifacts). Additionally, the algorithm does not account for motion artifacts. The corrected PNPA is obtained from the uncorrected PNPA by excluding artifact regions, according to the formula above. Artifact areas were manually identified via cross-sectional and en face image (orthoplane) review—Fig. 4 shows an example of such orthoplane data—an approach for artifact detection further discussed in *Part III*. The uncorrected and corrected PNPAs are shown in Fig. 3B,C; as expected, the presence of low-OCT-signal artifacts generates false positive non-perfused areas, which substantially change the uncorrected PNPA. This example demonstrates that detection and exclusion of artifacts is critical to obtaining valid quantitative OCTA measurements.

#### Part III: Detection and management of low-OCT-signal artifacts in widefield OCTA

Having established the causes and effects of low-OCT-signal artifacts, we now address their detection and management. Implementing detection and management procedures is important for obtaining accurate non-perfusion measurements, and thereby increasing measurement repeatability.

### Detection of low-OCT-signal artifacts

As illustrated in the previous section (Fig. 3A), low-OCT-signal artifacts can be difficult to detect using *en face* OCTA alone. Below we describe three approaches that use the underlying OCT data to identify low-OCT-signal artifacts; later, we discuss automatic low-OCT-signal artifact detection.

**The cross-sectional approach to artifact detection:** In the cross-sectional approach, OCT data are reviewed on a B-scan-by-B-scan basis, or “fly-through.” Regions of low OCT signal are clearly visible on OCT B-scans, and, if segmentation lines are overlaid on the B-scans, cross-sectional image review also allows segmentation errors to be easily identified. A limitation of the cross-sectional approach is that careful B-scan-by-B-scan analysis is often prohibitively time consuming because volumetric data are composed of hundreds of B-scans. However, as discussed in *Part IV*, a rapid B-scan fly-through can be useful for detecting gross errors. An additional limitation of cross-sectional review is that it does not permit direct comparison of any two regions which are not contained on the same B-scan. Thus, it can be difficult to obtain a global understanding of the data, or to compare the relative OCT signal strengths across different regions of the volume. Nevertheless, a B-scan fly-through is perhaps the simplest and most reliable approach for identifying general artifacts in volumetric OCT data sets.

**The En Face approach to artifact detection:** In the *en face* approach, OCT data are reviewed by inspecting an *en face* projection of the OCT data. In general, the *en face* OCT projection could be formed using different contours than those used to form the *en face* OCTA image. However, using the same contours facilitates detection of segmentation artifacts and is thus the approach chosen in this study.

As with the cross-sectional approach, regions of low OCT signal on the *en face* OCT image are straightforward to identify. Segmentation artifacts caused by low OCT signals, however, are more subtle: although these artifacts are ultimately caused by low OCT signal, they do not necessarily appear as low OCT signal on an *en face* OCT image. In particular, because an *en face* OCT image is formed by projecting data between two segmented contours (see Fig. 2), segmentation errors can cause inclusion of voxels that are outside of the layers of interest, or exclusion of voxels that are inside the layers of interest. Therefore, segmentation errors can produce widely varying (high, low, and everything in between) OCT signals in the corresponding *en face* OCT image. However, segmentation artifacts do have a common feature: they generate an abrupt signal change in the *en face* OCT image. In particular, moving from a region of correct segmentation to a region of incorrect segmentation means the voxels that are included (or excluded) in the projection change abruptly, thereby generating a sharp discontinuity in the *en face* OCT image. An example of *en face* segmentation artifact detection is shown in Fig. 4.

The advantage of the *en face* approach for artifact detection is threefold: first, an entire volume can be summarized in a single *en face* image, greatly expediting the review; second, a global view of the data set can be formed, allowing data from different B-scans to be compared; and third, since lesions are often identified using *en face* OCTA, the *en face* approach naturally facilitates identification of artifacts on/around the lesion of interest. The primary disadvantage of *en face* analysis is that, unlike cross-sectional analysis, it can be difficult to distinguish between segmentation versus thresholding artifacts. A second disadvantage of the *en face* approach is that, compared to the cross-sectional approach, it is relatively complex. A third disadvantage, as discussed in the following *Managing Detected Artifacts* section, is that the *en face* approach does not facilitate correction of segmentation errors.

**The orthoplane approach to artifact detection:** The orthoplane approach, which is commonly used for reviewing volumetric computed tomography (CT) and magnetic resonance imaging (MRI) data in radiology, is a combination of the cross-sectional and *en face* approaches. General orthoplane visualization involves viewing data along three orthogonal planes in a registered manner; however, due the unavailability of this option on some commercial systems, we restrict our attention to using a combination of a single *en face* OCT image (as in the preceding section) and the set of corresponding OCT B-scans. In particular, the orthoplane approach uses the *en face* approach to flag regions-of-interest—namely regions of low OCT signal, or regions having abrupt changes in the OCT signal—and then uses the cross-sectional approach to analyze these regions-of-interest. Compared to the *en face* approach, orthoplane review allows segmentation artifacts to be distinguished from thresholding artifacts, and, as discussed in the following *Managing Detected Artifacts* section, also facilitates correction of segmentation errors. Compared to the cross-sectional approach, the initial *en face* identification of suspicious regions greatly reduces the amount of data that must be cross-sectionally analyzed, thereby expediting the process. An example of orthoplane artifact detection is presented in Fig. 4.

**Automating artifact detection:** Although the *en face* and orthoplane approaches are much faster than the cross-sectional approach, manual image review remains undesirable in many settings. Moreover, manual approaches are inherently influenced by grader variability—what qualifies as “low signal” to one grader may not to another. For these reasons, it is important to develop automated approaches for artifact detection. In this study we present a simple, proof-of-concept algorithm to automatically detect widefield OCTA artifacts.

**Figure 5:**
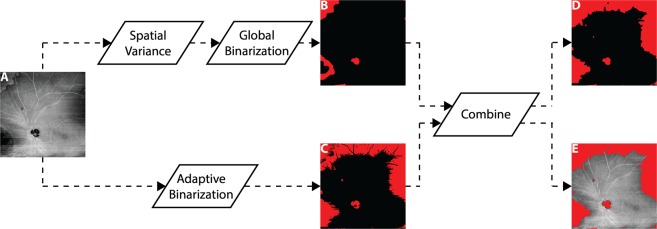
Workflow for automatic detection of low-OCT-signal artifacts. (**A**) Input *en face* OCT image. (**B**) Spatial variance mask to detect segmentation errors, formed by computing the spatial variance of the OCT image (9 pixel × 9 pixel kernel), and then binarizing the resulting variance image using an empirical threshold determined from the qualitative observations of a single grader. Additional morphological steps (closing and erosion) are used to remove spurious regions. (**C**) Low OCT signal mask, formed by an adaptive binarization of the input OCT image. In particular, a dynamic threshold (3 pixel × 3 pixel kernel) is computed relative to the mean intensity of the input *en face* OCT image. (**D**) Output artifact mask, formed by combing the masks of panels (B,C); again, morphological processing (erosion) is used to remove spurious regions. (**E**) Overlay of the output artifact mask on the input *en face* OCT image.

**Figure 6:**
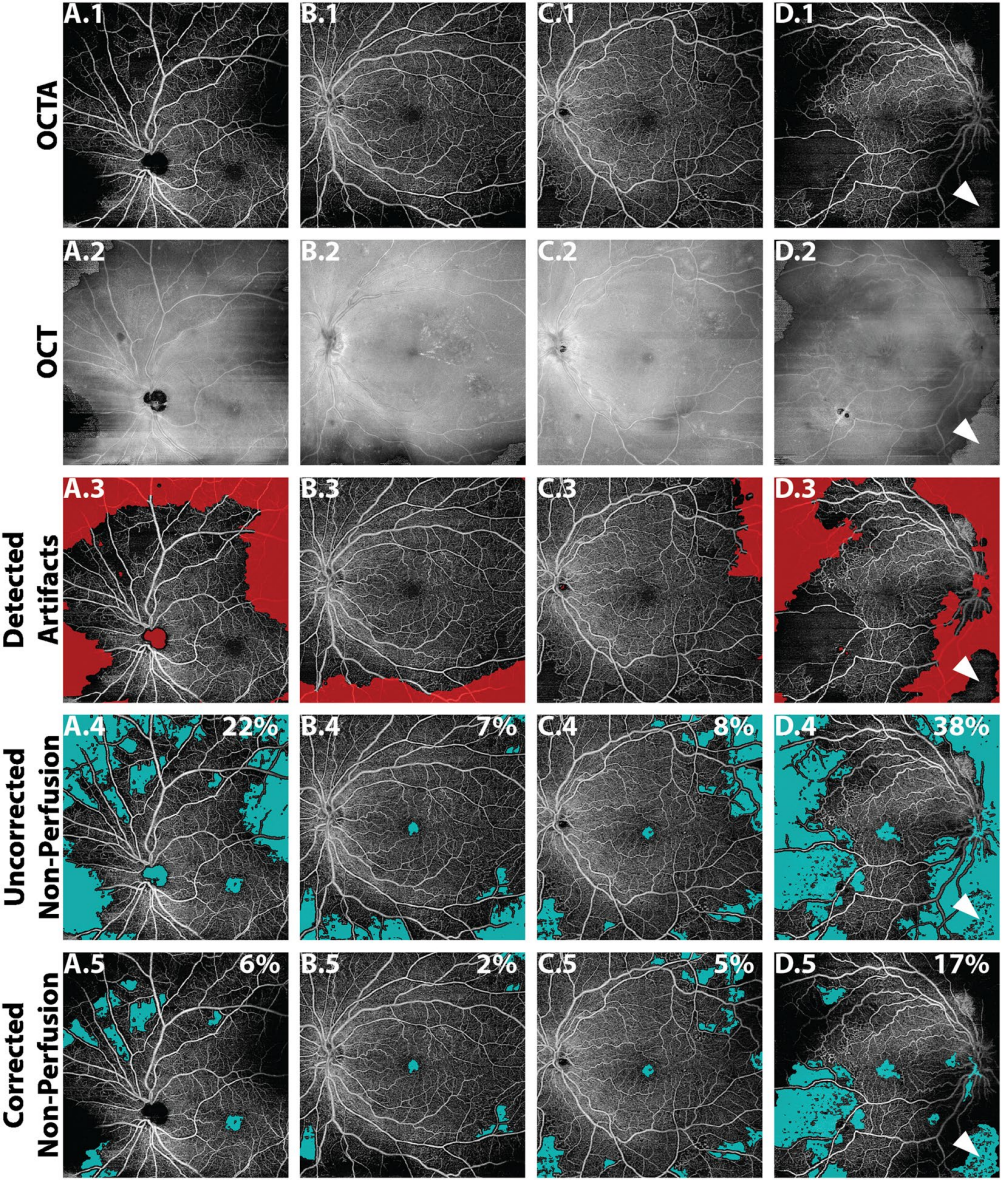
Examples of automatic artifact detection. All images are full retinal projections of 12 mm × 12 mm fields. Column (**A**) 68 year-old with mild NPDR; same as eye as in Fig. 3. Column (**B**) 74 year-old with severe NPDR. Column (**C**) 54 year-old with severe NPDR. Column (**D**) Right eye from same patient as column (**C**) PDR. Row 1: *En face* OCTA image of retinal vasculature. Row 2: Corresponding *en face* OCT image. Row 3: Automatically detected artifacts shown in red. Row 4: Uncorrected non-perfusion areas. Row 5, corrected non-perfusion areas. PNPAs are listed in the top-right corner of each panel in Row 4 and Row 5. The white arrowhead in column (**D**) points to a region that was incorrectly classified by the algorithm as being artifact-free (i.e., false negative).

With reference to Fig. 5, the automatic algorithm uses the *en face* approach to detect low-OCT-signal artifacts. The *en face* approach was selected rather than the orthoplane or cross-sectional approaches because of the ease with which *en face* data can be exported from commercial systems (most systems do not facilitate exportation of full volumetric data sets). Our algorithm detects low-OCT-signal artifacts using a two-fold, parallel strategy of identifying (1) regions having OCT signals with high spatial variance, and (2) regions of low OCT signal. The rationale for identifying regions of high spatial variance is that, as discussed previously, they are likely to correspond to segmentation artifacts. The rationale for identifying regions of low OCT signal is their association with thresholding artifacts—and, sometimes, with segmentation artifacts. Thus, by combining the two approaches, the algorithm detects both segmentation and thresholding artifacts. Examples of automatic artifact detection are shown in Fig. 6. PNPAs were computed using the algorithm described in *Part II*.

### Managing detected artifacts

Having discussed approaches for detecting low-OCT-signal artifacts, we now discuss how to manage these artifacts. From our discussion so far, it is clear that thresholding artifacts from low OCT signals are intrinsic to the data, whereas segmentation artifacts caused by regions of low OCT signals are attributable, at least immediately, to image processing (segmentation) errors. For thresholding artifacts, there is no possibility of “correcting” the data—the underlying signal is simply too low—and so the best mitigation strategy is to exclude the affected regions from the analysis. For segmentation artifacts, there is a *possibility* for correction—namely, by correcting the segmentation boundaries, either manually or automatically. However, correction of the segmentation boundaries does not always correct the artifact: it is possible that, if the OCT signal is too low in that region, correcting the segmentation boundaries merely converts the artifact from a segmentation artifact into a thresholding artifact. In this case, exclusion of the affected region is the best option.

The three detection approaches—cross-sectional, *en face*, and orthoplane—have different strengths and weaknesses for managing detected artifacts. Cross-sectional review is well suited to segmentation correction, but is ill-suited to exclude thresholded regions, which extend along the *en face* dimension. Conversely, *en face* review is well suited to exclude thresholded regions, but does not, by itself, allow for segmentation correction. Again, orthoplane review seems to be a good compromise: cross-sectional inspection facilitates segmentation correction, while *en face* inspection facilitates exclusion of thresholding artifacts.

### A systematic, codified approach to artifact detection and management

**Figure 7:**
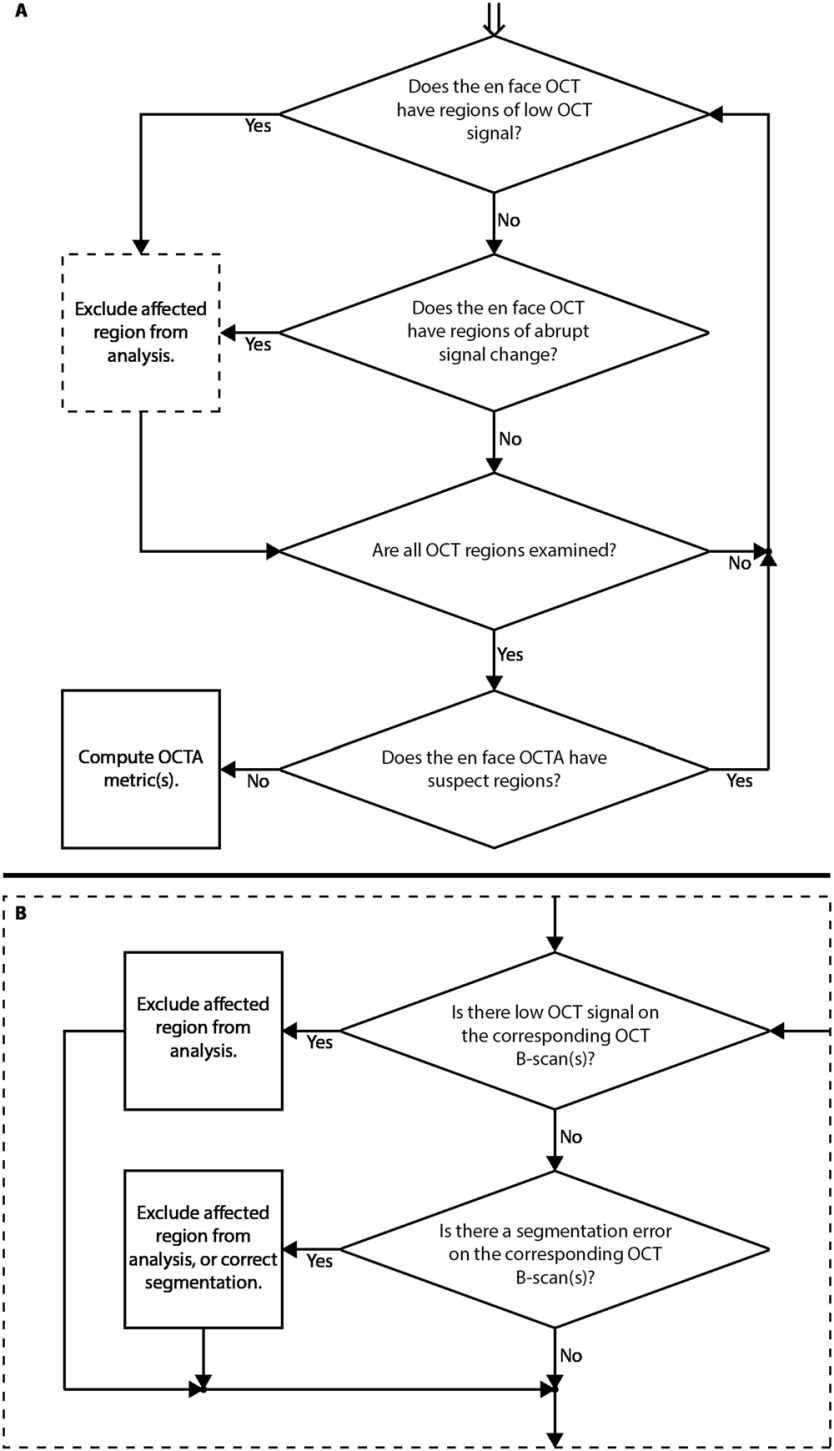
Workflows for the *en face* and orthoplane approaches to low-OCT-signal artifact detection. (**A**) Workflow for *en face* artifact detection; workflow begins at the top of the diagram, with the double arrow. In the *en face* approach, segmentation artifacts cannot be distinguished from thresholding artifacts; as such, detected artifacts are excluded from the analysis (dashed box of panel A). (**B**) In the orthoplane approach, the dashed box of panel (A) is substituted for a cross-sectional analysis of the region-of-interest. Cross-sectional analysis can be used to differentiate segmentation artifacts from thresholding artifacts, and possibly correct the former by adjusting the segmentation.

As the prior sections have shown, detection and management of artifacts is relatively subtle. Therefore, it is helpful to approach artifact detection using a systematic, codified scheme. Figure 7 presents flow charts for the *en face* and orthoplane approaches to artifact detection and management. The flow charts highlight that the *en face* and orthoplane approaches are similar, with the difference arising from how suspect regions in the *en face* OCT image are managed (dashed box of Fig. 7). While the determination of low-OCT-signal artifacts is ultimately made using OCT data, it is good practice to also examine the *en face* OCTA image for suspicious regions (e.g., signal discontinuities, or un-natural regions of low OCTA signal); these suspicious regions can then be investigated on OCT, as shown by the iterative loop present in Fig. 7A.

#### Part IV: reducing low-OCT-signal artifacts through optimal instrument alignment

Having discussed methods for managing low-OCT-signal artifacts, we now briefly discuss suggested best-practices for acquiring artifact-free widefield OCT(A) data. These best-practices are also useful for generally improving signal quality and for avoiding other common artifacts—for example, “mirror artifacts,” which occur when the retina crosses the zero delay position, causing the retinal image in the B-scan to fold over on itself.

### Patient preparation

1. Patient Instruction: inform the patient: how long the OCT(A) scan will take; that they should try not to move, blink, or change their focus during the scan; and that before imaging begins you will align the system, which may take some time, during which they are free to blink as desired. Some commercial instruments which use eye tracking can acquire data during eye blinks, which reduces corneal drying and patient fatigue, so the instruction not to blink should be amended accordingly.
2. Pupil Dilation: because smaller pupil sizes exacerbate vignetting, pupil dilation is helpful for imaging, particularly for wider fields-of-view.

### Patient Imaging

1. Patient Seating: ensure that the patient is comfortably seated, with their head in a stable position; this may require adjusting the table height and/or head-rest position. If possible, have the patient place their forearms on the table for stability (some systems have hand-grips for this purpose). Because alignment and imaging can take several minutes, it is important for the patient to be in a comfortable position that they can maintain throughout imaging.
2. Fixation Target: ask the patient to focus on the fixation target. If the fixation target is large, or has several components, it may be helpful to instruct the patient where on the target they should focus (e.g., “on the center of the cross” or “on the central dot”). In some systems, light from other sources may be visible to the patient—for example, from a tracking line-scan laser ophthalmoscope, camera illumination, or the OCT beam itself. As such, you may want to ask the patient to try their best not to look at this additional light. We have found that light from the OCT beam can be especially distracting, with patients often “following” the OCT beam as it scans along the slow-scan axis (this is particularly a concern with shorter wavelength, 840 nm systems, where the OCT beam is more likely to be visible).
3. System Alignment: adjust the instrument transverse position and working distance until the patient’s retina is visible in the preview B-scans. If the retina appears tilted (i.e., the retina is closer to the top on one side of the B-scan), adjust the OCT beam pivot position on the pupil by adjusting the transverse or vertical position of the system. In some cases, the macula may be tilted and it will not be possible to achieve an alignment where the retina appears flat. Some instruments provide a preview of the top and bottom scans in the field-of-view; in this case, the instrument should be aligned so that the retina is well positioned in both the top and bottom scans. Note that high signal strength throughout the B-scan is indicative of good alignment between the beam pivot and pupil plane (less likely that there will be vignetting), whereas lower signal strength, particularly on the edges of a B-scan, indicates poor alignment between the beam pivot and pupil plane (more likely that there will be vignetting). In the latter case, adjust the system’s working distance (moving the system closer to, or farther from, the patient) until good signal strength is observed throughout the B-scan. If the system has an integrated fundus camera, maximizing the viewable region on the fundus image can also be useful for alignment. Some systems also have guided alignment prompts which can be used to set the instrument working distance.
4. Reference Arm Positioning: the axial (depth) position of the retinal image on the B-scan is determined by a combination of the working distance and reference arm (zero delay) position. In particular, with the working distance held fixed, adjusting the reference arm position moves the retinal image up/down within the B-scan. Although adjusting the working distance also moves the retinal image up/down within the B-scan, as we saw in Step 3, changing the working distance alters the alignment between the OCT beam pivot and pupil plane. In contrast, the alignment of the beam pivot with the pupil plane is independent of the reference arm position, meaning that the reference arm position—not the working distance—should be used to adjust the axial position of the retinal image in the B-scan.
  In general, the retina should be positioned at a distance close to, but not overlapping with, the zero delay position. In many OCT systems and imaging configurations, the zero delay is positioned in front of the retina, meaning that mirror artifacts occur when the retinal image reaches the top of the B-scan. In other configurations, such as enhanced depth imaging, the zero delay is positioned behind the retina, meaning that mirror artifacts occur when the retinal image reaches the bottom of the B-scan. Focusing on the former imaging configuration, which is more typical for imaging of retinal vasculature, it is desirable to adjust the reference arm position so that the retinal image is in the upper portion of the B-scan, leaving some gap (i.e., vitreous) between the top of the retinal image and the top of the B-scan. This gap between the top of the retinal image and top of the B-scan prevents the retina from crossing the zero delay during imaging, which would generate a mirror artifact. The desired size of the gap varies with different fields-of-view and retinal curvatures; however, a typical rule-of-thumb is to leave a gap of 1/5th of the axial dimension of the B-scan. Because wider fields-of-view encompass more retinal curvature (i.e. more variation of the axial position of the retina), the wider the field-of-view, the larger the recommended gap. Correct positioning of the retinal image within the B-scan is a balance between leaving too small of a gap (which risks mirror artifacts), and too large of a gap (which risks reduced signal due to sensitivity roll-off). Many systems have an automatic function for optimizing the reference delay.
5. Polarization Optimization: some systems include an option to automatically match the polarization of light from the reference arm to light backscattered from the patient’s eye in order to maximize the OCT signal. Since only light with the same polarization can interfere, strong OCT signal requires well-matched polarization.
6. Iteration: repeat steps 3–5 as needed.
7. Patient Instruction: inform the patient before you initiate imaging. We find it helpful to provide a prompt that allows the patient to prepare for imaging (e.g., “imaging in 3…2…1…don’t move, don’t blink”). When the scan is completed, inform the patient.

### Rapid Data Review

1. Reviewing B-scan OCT Data: B-scans should be reviewed immediately following an acquisition for evidence of vignetting, mirror artifacts, and other abnormalities. This post-acquisition review is not intended to be comprehensive, but rather to detect any gross artifacts so that the scan can be re-acquired if needed. We have found that a quick 10–15 second B-scan fly-through is usually sufficient for this purpose (some systems also display B-scans on the screen during the acquisition itself, which can be useful for identifying mirror artifacts). Evidence of mirror artifacts and/or substantial vignetting is typically grounds for re-acquiring the scan.

A special note is merited for systems (e.g., Avanti, Optovue, Inc., Fremont, CA) that form the final output OCT(A) image by registering and merging 2 or more volumes acquired in separate acquisitions (e.g., an X-fast and Y-fast scan): if even one of the input volumes is corrupted by an artifact (with the exception of motion artifacts), the final output image will likely also be corrupted. Moreover, when possible, the 2 or more volumes should be acquired without realignment of the instrument/patient between acquisitions—realignment increases the probability that the volumes will not register and merge correctly. Similarly, it is also important that the patient’s (side-to-side) head tilt, which determines the rotation of the retina, does not change between the repeated volume acquisitions.

2. Reviewing *En Face* OCT Data: As discussed in this paper, *en face* OCT data can also be useful for detecting regions of low OCT signal. However, it is typically impractical to carefully examine *en face* data (e.g., using the workflow of Fig. 7) during an imaging session. Nevertheless, a quick inspection of a full *en face* OCT projection can sometimes reveal gross artifacts—though usually such artifacts are also apparent on a B-scan fly-through.
3. Reviewing OCTA Data: Although it is potentially useful, rapid assessment of OCTA data can be complicated and misleading. In particular, as explained in this paper, it can be difficult to correctly interpret OCTA features (such as regions of low OCTA signal) from OCTA data alone. Therefore, we recommend caution when using OCTA data in real time during an imaging session to determine the presence of artifacts in a scan.

## Discussion

OCTA has provided clinicians and researchers with a new tool for viewing and quantitating ocular vasculature. However, compared to dye-based imaging, early commercial OCTA systems offered only a fraction of the field-of-view. Thus, the introduction of widefield OCTA is a most welcome advance. At the same time, OCTA suffers from a variety of artifacts^13,21^, and, as outlined in *Part I* and *Part II* of this study, a subset of these artifacts are exacerbated by larger fields-of-view. As stated in *Part I*, for moderate fields-of-view, such as the 12 mm × 12 mm fields of this study, vignetting is the predominant cause of low-OCT-signal artifacts in widefield OCT(A). However, demonstrations of ultra-widefield OCT^12,22,23^ suggest that commercial OCTA fields will continue to increase and, consequently, contributions from other causes of low OCT signal will become increasingly important. Nevertheless, the analysis and methods of *Part II* and *Part III* of this study are equally applicable for all causes of low OCT signal, and therefore provide a general scheme for interpreting low-OCT-signal artifacts in widefield OCTA.

**Figure 8:**
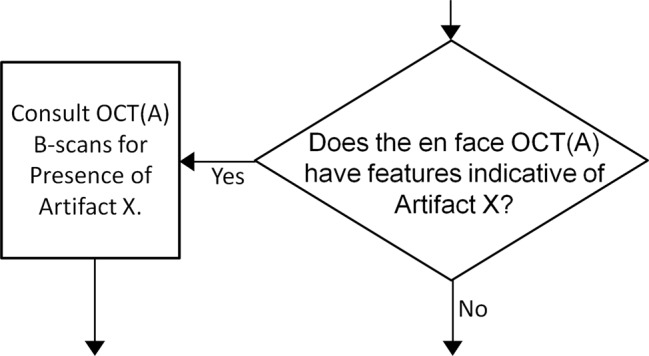
General approach to rapid identification of artifacts using orthoplane viewing. Artifact X represents an artifact that appears in the *en face* data.

In this study we presented three different approaches—cross-sectional, *en face*, and orthoplane—for detecting low-OCT-signal artifacts. A summary of the advantages and limitations of the three methods is provided in Table 2. In our opinion, the orthoplane approach combines the advantages of the cross-sectional and *en face* approaches, and is therefore our recommended method for detecting low-OCT-signal artifacts. A limitation of the orthoplane approach is that it requires full access to the OCT volume—that is, B-scans and *en face* data—in a co-registered manner, which, while supported by the system used in this study, may not be available on all commercial systems. In addition to detecting low-OCT-signal artifacts, the orthoplane approach to data review, codified in Fig. 7, provides a framework for detecting and interpreting other OCT(A) artifacts. Indeed, with reference to Fig. 8, the orthoplane approach is well suited to rapid detection of any artifact that is manifest the *en face* OCT(A) image. Because of this, we believe that orthoplane OCT(A) data review is one of the best approaches for to minimizing artifact-related misinterpretations of all types. In particular, the orthoplane approach is useful for detecting many types of segmentation errors, not just those caused by low OCT signal. Since segmentation is a critical image processing step in generating *en face* OCT(A) data, and because segmentation can be compromised by many factors (e.g., the presence of pathology), techniques for detecting and correcting segmentation errors are of utmost importance.

**Table 2:** Advantages and disadvantages of the three approaches to artifact detection.

	Cross-Sectional	*En Face*	Orthoplane
Fast	×	✓	✓
Gives global view of data	×	✓	✓
Allows differentiation of thresholding and segmentation artifacts	✓	×	✓
Facilitates correction of segmentation artifacts	✓	×	✓
Only requires access to *en face* data	×	✓	×
Simple	✓	×	×

In addition to manual approaches, we presented an automatic algorithm for detecting low-OCT-signal artifacts. As noted in *Part III* of this paper, the choice of automating the *en face* approach, rather than the cross-sectional or orthoplane approaches, was made because *en face* data were readily exportable from the commercial instrument used in this study. However, since an automatic algorithm can examine hundreds of B-scans in a matter of seconds—or less—the drawbacks of cross-sectional review are greatly mitigated by automation. Additionally, with access to segmentation contours, automatic algorithms could be designed to detect segmentation artifacts directly—for example, by detecting abrupt changes in the contours.

It should be noted that the algorithm developed in this paper was preliminary, and intended as a proof-of-concept. As such, we made no attempts to validate the algorithm against expert assessment. Indeed, even on the four tested cases (Fig. 6), we can notice limitations: (1) the detection of the optic nerve head as a low-OCT-signal artifact; (2) the erroneous false-negative shown in column D (white arrowhead); and (3) the discrepancy between the automatically corrected PNPA measurement of 6% (for the case of Fig. 6A.5) versus the manually corrected PNPA measurement of 13% for the same case (Fig. 3C). It is noteworthy that the latter limitation may not be a limitation at all: vignetting-induced low OCT signals may produce a continuum of effects, from only a slight reduction of the full OCT signal to no OCT signal at all. As such, there is typically no clear delineation between regions of low OCT signal and regions of sufficient OCT signal; rather, the grader uses their judgment to demarcate a line of transition. Thus, we would expect that different graders would choose different delineations, resulting in different excluded regions. Although our automatic algorithm did not choose the same delineation as the manual grader—it was not trained to do so—it would nevertheless be consistent in its choice, which is one of the advantages of automatic artifact detection.

Low-OCT-signal artifacts are not the only artifacts whose incidence and severity increase with larger fields-of-view. For example, all else being equal, larger field sizes result in reduced A-scan sampling densities, which can cause capillaries to be missed/combined, and, as a result, non-perfusion area measurements to be altered. Like low-OCT-signal artifacts, sampling artifacts can cause artifactual scan-to-scan variations in non-perfusion area measurements—that is, if the same eye is imaged repeatedly, the non-perfusion area measurements may be inconsistent. As another example, magnification artifacts caused by axial eye length variations can affect area measurements^24^, producing inter-eye variations—that is, two eyes with equal non-perfusion areas will produce inconsistent measurements. Finally, distortions in mapping the curved retinal surface to a flat *en face* image cause uncorrected area measurements to deviate from true areas^25^. To first order, this creates a constant bias, or offset, from the true non-perfusion area; more accurately, noting that more peripheral non-perfusion areas are underestimated, this mapping can also cause eye-to-eye variations (assuming that the spatial distribution of the non-perfusion areas is unequal). Table 3 presents a summary of some common artifacts relevant to widefield OCTA, and their effects on measurement variability. Minimizing these artifacts, particularly those that cause scan-to-scan or eye-to-eye variations, is important when incorporating non-perfusion measurements into clinical trials.

**Table 3:** Common widefield OCT artifacts and their variation types.

Artifact Type	Variation Type
Low-OCT-Signal	Scan-to-scan variation
Reduced Sampling Density	Scan-to-scan variation
Magnification	Eye-to-eye variation
Field-of-View Mapping	Constant bias/eye-to-eye variation

A limitation of this study is that we have used a small set of cases, from a single device, acquired at a single imaging center. However, the aims of this study—to outline causes and effects of, solutions to, and prevention of, widefield OCTA artifacts—are not substantially impacted by this limitation: (1) vignetting, the predominant cause of low-OCT-signal artifacts in widefield OCT(A), is fundamental to any beam scanning system, which, to the best of knowledge, encompasses every present—and past—commercial ophthalmic OCT system; (2) the effects of low OCT signal on the OCTA signal are also shared by all OCTA systems because regions of low OCT signal produce regions of low OCTA signal; and (3) the approaches for detecting low-OCT-signal artifacts, are, by the merits of (2), general to all OCTA systems. In fact, as discussed, the approaches presented in this paper are extendable to other artifact types. For these reasons, the restricted number and sources of cases does not diminish the conclusions of the study. Another limitation of the study is that we only quantitatively examined the effects of low-OCT-signal artifacts using a single metric (PNPA), on a single disease type (DR), and on a single vascular region (retinal vasculature). While this limits the extrapolation of effect sizes to different metrics, diseases, and plexuses, PNPA in the retinal vasculature of diabetic eyes represents a useful and interpretable model for the behavior of other metrics and other vascular alterations.

## Conclusion

In this study we investigated low-OCT-signal artifacts in widefield OCTA. In particular, we provided a physical explanation of why vignetting, the predominant cause of low signal, is exacerbated by larger fields-of-view. We then showed how artifacts produced by regions of low OCT signal propagate through the processing pipeline to produce artifacts in the final OCTA image, and provided a set of approaches for mitigating these artifacts. Finally, we described suggested best-practices for widefield OCTA imaging, with an emphasis on preventing artifacts. Understanding, prevention, and management of artifacts in widefield OCTA is critical to achieving reproducible quantitative measurements, and, consequently, will be integral to longitudinal studies using quantitative OCTA to assess disease progression and/or response to therapy.

## Data Availability

All data generated and/or analyzed during the present study are available from the corresponding author on reasonable request.
